# Age and mean platelet volume-based nomogram for predicting the therapeutic efficacy of metoprolol in Chinese pediatric patients with vasovagal syncope

**DOI:** 10.1007/s12519-024-00802-5

**Published:** 2024-04-13

**Authors:** Xiao-Juan Du, Ya-Qian Huang, Xue-Ying Li, Ying Liao, Hong-Fang Jin, Jun-Bao Du

**Affiliations:** 1 Department of Pediatrics, Peking University First Hospital, No. 1 Xi’anmen Street, West District, Beijing, 100034 China; 2 State Key Laboratory of Vascular Homeostasis and Remodeling, Peking University, No. 38, Xueyuan Road, Haidian District, Beijing, 100191 China; 3 Department of Statistics, Peking University First Hospital, Beijing, 100034 China

**Keywords:** Metoprolol, Nomogram, Retrospective study, Therapeutic effect, Vasovagal syncope

## Abstract

**Background:**

Vasovagal syncope (VVS) is the most common type of orthostatic intolerance in children. We investigated whether platelet-related factors related to treatment efficacy in children suffering from VVS treated with metoprolol.

**Methods:**

Metoprolol-treated VVS patients were recruited. The median duration of therapy was three months. Patients were followed and divided into two groups, treament-effective group and treatment-ineffective group. Logistic and least absolute shrinkage selection operator regressions were used to examine treatment outcome variables. Receiver-operating characteristic (ROC) curves, precision–recall (PR) curves, calibration plots, and decision curve analyses were used to evaluate the nomogram model.

**Results:**

Among the 72 patients who complete the follow-up, treatment-effective group and treatment-ineffective group included 42 (58.3%) and 30 (41.7%) cases, respectively. The patients in the treatment-effective group exhibited higher mean platelet volume (MPV) [(11.0 ± 1.0) fl vs. (9.8 ± 1.0) fl, *P* < 0.01] and platelet distribution width [12.7% (12.3%, 14.3%) vs. 11.3% (10.2%, 12.2%), *P* < 0.01] than those in the treatment-ineffective group. The sex ratio was significantly different (*P* = 0.046). A fit model comprising age [odds ratio (OR) = 0.766, 95% confidence interval (CI) = 0.594–0.987] and MPV (OR = 5.613, 95% CI = 2.297–13.711) might predict therapeutic efficacy. The area under the curve of the ROC and PR curves was computed to be 0.85 and 0.9, respectively. The *P* value of the Hosmer–Lemeshow test was 0.27. The decision curve analysis confirmed that managing children with VVS based on the predictive model led to a net advantage ranging from 0.01 to 0.58. The nomogram is convenient for clinical applications.

**Conclusion:**

A novel nomogram based on age and MPV can predict the therapeutic benefits of metoprolol in children with VVS.

## Introduction

Vasovagal syncope (VVS) is a common type of orthostatic intolerance in the pediatric population [1]. Although the prognosis is regarded as benign, injuries associated with syncope may occur in 33% of patients with VVS [2]. Recurrent syncope may lead to not only physical trauma but also mental disorders and poor quality of life in affected children [3, 4]. Unfortunately, no pharmacological treatment with high-quality evidence has been proven to be effective for treating pediatric VVS. Metoprolol is a widely used type of β-adrenergic receptor blocker and is commonly used for treating pediatric VVS [5]. The possible mechanisms for the action of β-blockers in treating patients with VVS include antagonizing increased sympathetic nerve activity [6] and/or the elevated levels of circulating epinephrine (EP) and norepinephrine (NE) [7]. However, the therapeutic effect of metoprolol in pediatric VVS patients is unsatisfactory [8]. This phenomenon may be explained by the fact that not all children with VVS exhibit increased sympathetic activity. Therefore, biomarkers that represent the sympathetic activation status are urgently needed to predict the therapeutic efficacy of metoprolol. According to a mechanism-based study, sympathetic stimulation may boost the binding, movement, and formation of proplatelets in megakaryocytes [9]. The mean platelet volume (MPV) has been reported to be closely associated with enhanced sympathetic nerve activity in VVS [10]. Therefore, this study aimed to determine whether platelet-related parameters could predict the outcomes of metoprolol therapy in children with VVS.

## Methods

### Research design and subjects

This was a retrospective study that included children aged 4–17 years with VVS. The study was approved by the Institutional Ethics Committees of Peking University First Hospital (No. 202122496), and informed consent was obtained from the patients and their parents.

We included patients based on the following criteria: patients (1) were diagnosed as VVS and were admitted to the pediatric department of Peking University First Hospital between April 2002 and September 2022 and (2) were treated with metoprolol. We excluded patients based on the following criteria: patients (1) had not experienced any syncope or presyncope attack three months before the admission; (2) had other comorbidities, including cardiogenic, neurologic, hematologic, inflammatory, or metabolic diseases; (3) received other medications, including midodrine hydrochloride and pyridostigmine; and (4) did not receive a continuous treatment, or those whose treatment duration was less than one month.

VVS was diagnosed based on the following established criteria [11, 12]: (1) syncope episodes often occur in response to various factors that increase a patient's vulnerability, including transitioning from a lying to a standing position, prolonged periods of standing, and exposure to hot and humid conditions; (2) recurrent syncope episodes; (3) a positive response during the head-up tilt test (HUTT); and (4) patients were excluded for other potential causes of fainting-like events, including epilepsy, hypoglycemia, and cardiac syncope.

### Treatment protocol and follow-up

All patients were administered 0.5 mg/kg/day of metoprolol. The median treatment duration was 3 (2, 3) months [13]. After starting the treatment, the therapeutic response was followed up by trained doctors over the telephone or through outpatient visits. The patients were categorized into two groups based on their therapeutic response. Effectiveness was defined as the non-recurrence of syncope during follow-up [14].

### Head-up tilt test

As previously described [11, 12, 15, 16], HUTT was performed in areas with dim lighting, warmth, and minimum noise levels. In the present study, the HUTT was performed around 9 a.m. in the morning for all participants. Patients were required to fast for at least four hours and halt the use of drugs that influenced the autonomic nervous system for a period equal to five half-lives of the respective medication. The subjects were monitored in the supine position on a tilt table for 20 minutes (SHUT-100A, Standard, Jiangsu, and ST-711, Juchi, Beijing, China). The tilt table was set at a 60-degree angle, and the test was continued until either a positive reaction occurred or 45 minutes had passed.

### Data collection

Demographic data (sex, age, height, and weight), symptoms (total attacks of syncope and duration), and baseline hemodynamics [heart rate, systolic blood pressure, diastolic blood pressure, the platelet-related variables platelet count (PLT), MPV, platelet crit (PCT), and platelet distribution width (PDW)] were analyzed. Demographic data and baseline hemodynamic data were collected from electronic medical records (Donghua and Kaihua, Beijing, China). Blood samples were taken in the morning on the same day or the day before the HUTT for all the participants. After at least 12 hours of fasting, venous blood was collected in a tube containing dipotassium ethylenediaminetetraacetic acid and measured using a Sysmex XE-5000 (Sysmex Corporation, Kobe, Japan). The time interval between blood sample collection and analysis was less than 1 hour for all participants. The platelet count was analyzed by the optical method (PLT-O). MPV was calculated by dividing the total mass of the platelets (PCT) by the total number of platelets.

### Data analysis

The data were analyzed using SPSS (version 26.0, IBM) and R (version 4.2.3). The normality of the distribution was tested by the Kolmogorov‒Smirnov method for continuous variables, among which normally distributed data are expressed as the mean ± standard deviation, while non-normally distributed data are expressed as the median (25th percentile, 75th percentile). The difference was computed using χ^2^ or Fisher's exact test for categorical variables, Student’s *t* test for normally distributed continuous variables, or the Mann‒Whitney *U* test for non-normally distributed data. Mean imputation was used to address six missing PCT data points [17, 18]. Least absolute shrinkage and selection operator (LASSO) regression (glmnet package) and logistic regression were used to construct the model. Decision curve analysis (DCA; rmda package), precision‒recall (PR; modEvA package) curves, calibration plots (MASS package), receiver-operating characteristic (ROC) curves, and nomograms (regplot package) were used to evaluate the model. The best cut-off value was established using the Youden index. *P* < 0.05 indicated statistical significance.

## Results

### Baseline characteristics

**Fig. 1:**
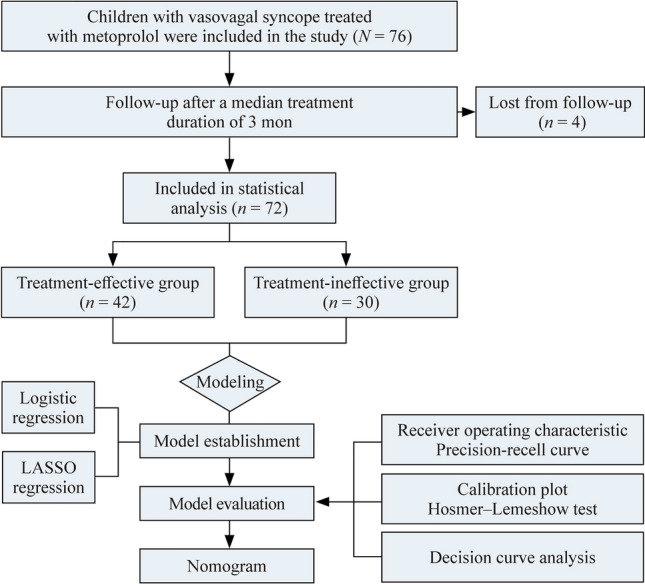
Flowchart for predicting the therapeutic efficacy of metoprolol in pediatric patients with vasovagal syncope. *LASSO* least absolute shrinkage and selection operator

Among the 76 VVS patients included, 72 (94.7%) completed the follow-up (Fig. 1). Thirty-four (47%) patients were females, and 38 (53%) were males. The median age of patients was 12 (10, 14) years. A comparison of baseline characteristics between the treatment-effective group (42 patients, 58%) and treament-ineffective group (30 patients, 42%) is shown in Table 1. MPV [(11.0 ± 1.0) fl vs. (9.8 ± 1.0) fl, *P* < 0.05] and PDW [(12.7% (12.3%, 14.3%) vs. 11.3% (10.2%, 12.2%), *P* < 0.05] were greater in the treatment-effective group than those in the treatment-ineffective group. In addition, a statistically significant difference in the sex ratio was found between the two groups (*P* = 0.046). Moreover, there were no significant differences in the other indicators (all *P* values > 0.05).

**Table 1:** Comparison of baseline characteristics between the treatment-effective and treatment-ineffective groups of children with vasovagal syncope and metoprolol therapy

Items	Total	Effective	Ineffective	*P*
Sex, male/female	38/34	18/24	20/10	0.046
Age (y)	12 (10, 14)	12 (9, 14)	13 (11, 14)	0.220
Weight (kg)	51 ± 16	49 ± 16	53 ± 15	0.237
Height (m)	1.6 ± 0.1	1.6 ± 0.2	1.6 ± 0.1	0.266
Duration of treatment (mon)	3 (2, 3)	3 (2, 3)	3 (2, 3)	0.995
Duration of history (mon)	10 (3, 36)	10 (4, 36)	14 (2, 36)	0.828
Duration of hospitalization (d)	8 (7, 10)	8 (7, 9)	9 (7, 11)	0.413
Total attacks of syncope (time)	3 (2, 5)	3 (2, 5)	4 (2, 5)	0.963
Hemodynamic phenotypes				0.964
Vasoinhibitory	53	31	22	
Cardioinhibitory + Mixed	19	11	8	
HR (bpm)	80 ± 12	80 ± 13	80 ± 12	0.839
SBP (mmHg)	111 ± 12	110 ± 12	112 ± 11	0.377
DBP (mmHg)	64 ± 7	64 ± 6	65 ± 7	0.477
PLT (× 10^9^/μL)	270 ± 60	260 ± 53	284 ± 67	0.095
MPV (fl)	10.5 ± 1.0	11.0 ± 1.0	9.8 ± 1.0	< 0.001
PCT (%)	0.28 (0.25, 0.31)	0.28 (0.25, 0.31)	0.28 (0.25, 0.32)	0.752
PDW (%)	12.4 (11.2, 12.9)	12.7 (12.3, 14.3)	11.3 (10.2, 12.2)	< 0.001

Data are presented as mean ± standard deviation or median (25th percentile, 75th percentile). *HR* heart rate, *SBP* systolic blood pressure, *DBP* diastolic blood pressure, *PLT* platelet, *MPV* mean platelet volume, *PCT* platelet crit, *PDW* platelet distribution width, *bpm* beat per minute, *fl* femtoliter.

### Construction of the predictive model

**Fig. 2:**
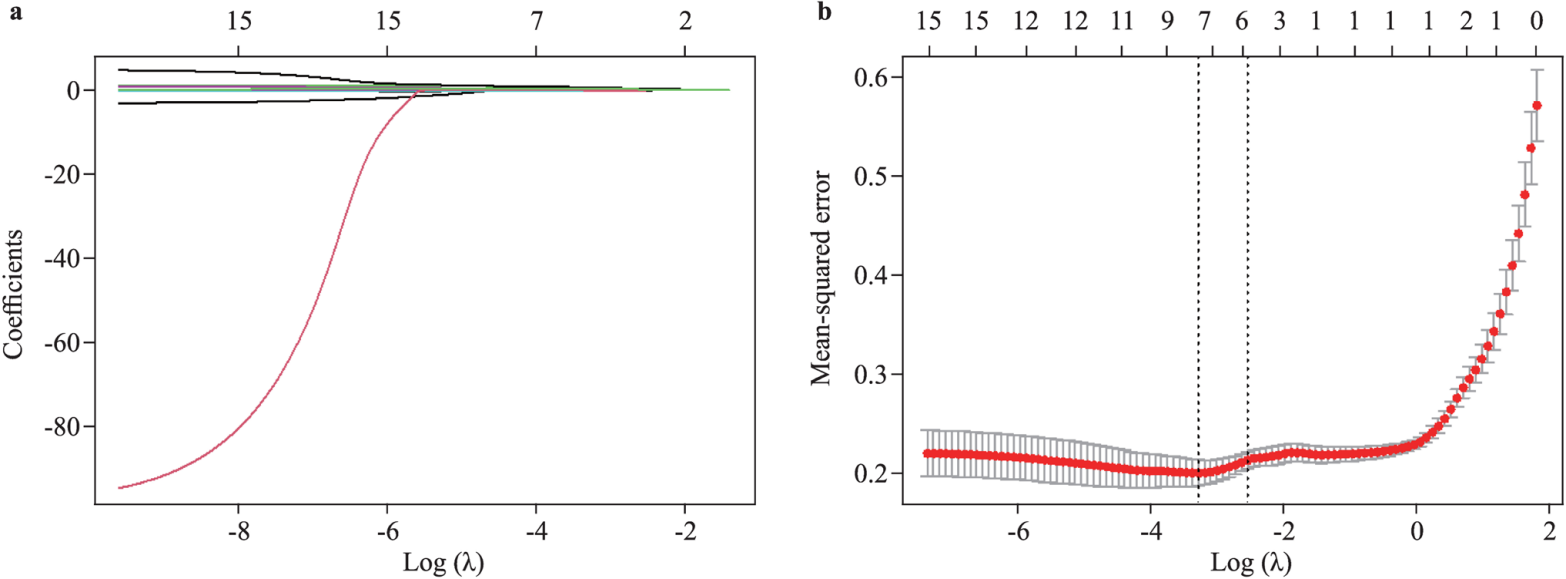
Feature selection by LASSO regression for predicting the therapeutic effect of metoprolol in pediatric patients with vasovagal syncope. **a** The *Y*-axis represents the coefficient. The log of the parameter λ is shown on the *X*-axis. Variables are represented by colored curves. When the parameter λ reaches a certain value, some unimportant variable coefficients are compressed to 0, indicating that the variable has been removed from the model; **b** the *Y*-axis represents the mean-squared error (MSE). The log of the parameter λ is shown on the *X*-axis. The left dotted line represents the λ at which the minimal MSE is achieved. The right dotted line represents the λ at which one standard error of the minimal MSE is achieved. In this study, five variables are selected at the right dotted line. *LASSO* least absolute shrinkage and selection operator

All factors included in the study were subjected to LASSO regression for dimensional reduction processing. As shown in Fig. 2, five variables were chosen as potential candidates, namely, age, duration of hospitalization, sex, MPV, and PDW, while the coefficients of the other non-selected factors were reduced to zero with stringent penalization.

Variance inflation factors (VIFs) were calculated to detect multicollinearity among the candidates (Table 2). The aforementioned parameters were subjected to multivariate logical regression, as their VIFs were not more than 5. Finally, as shown in Table 3, age [odds ratio (OR) = 0.766, 95% confidence interval (CI) = 0.594–0.987] and MPV (OR = 5.613, 95% CI = 2.297–13.711) were found to be independent factors associated with metoprolol therapeutic efficacy.

**Table 2:** Results of multicollinearity analysis

Items	Tolerance	VIF
Age (y)	0.982	1.018
Duration of hospitalization (d)	0.881	1.135
Gender	0.958	1.044
MPV (fl)	0.244	4.104
PDW (%)	0.256	3.907

*VIF* variance inflation factor, *MPV* mean platelet volume, *PDW* platelet distribution width, *fl* femtoliter

**Table 3:** Results of multivariate logistic regression analysis

Items	*B*	SE	Wald	*P*	OR	95% CI	
						Lower	Upper
Age (y)	− 0.267	0.129	4.239	0.040	0.766	0.594	0.987
MPV (fl)	1.725	0.456	14.328	< 0.001	5.613	2.297	13.711
Constant	− 14.511	4.313	11.318	0.001	0.000		

*MPV* mean platelet volume, *SE* standard error, *OR* odds ratio, *CI* confidence interval, *fl* femtoliter

### Nomogram of the model

**Fig. 3:**
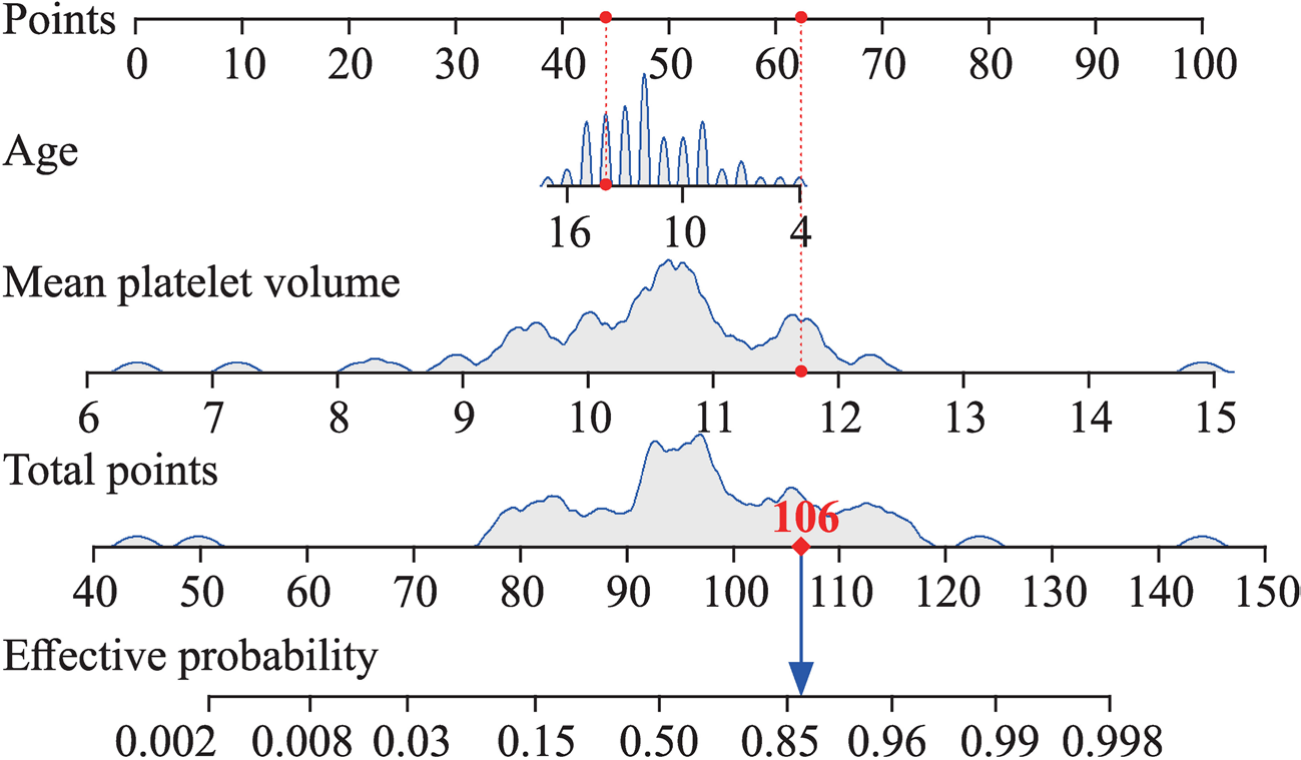
Nomogram of the model for predicting the therapeutic effect of metoprolol in pediatric patients with vasovagal syncope. For instance, if a patient is 14 years old and the mean platelet volume (MPV) is 11.7 femtoliter (fl), the corresponding scores, which are marked with red dots on the top horizontal line, can be acquired (44 points for age, 62 points for MPV). Therefore, the total number of points is approximately 106. A dot representing the total number of points corresponds to a probability of 0.87, which is marked by the blue arrow on the bottom horizontal line. Thus, metoprolol is recommended for use. Age is measured in year; and MPV is measured in fl

Based on logistic regression analysis, the regression equation was as follows: logit(*p*) = − 14.511 − 0.267 × age + 1.725 × MPV. A model containing age and the MPV was constructed to predict the therapeutic efficacy of metoprolol. The nomogram was used to visualize the findings of the model (Fig. 3).

### Evaluation of the model

**Fig. 4:**
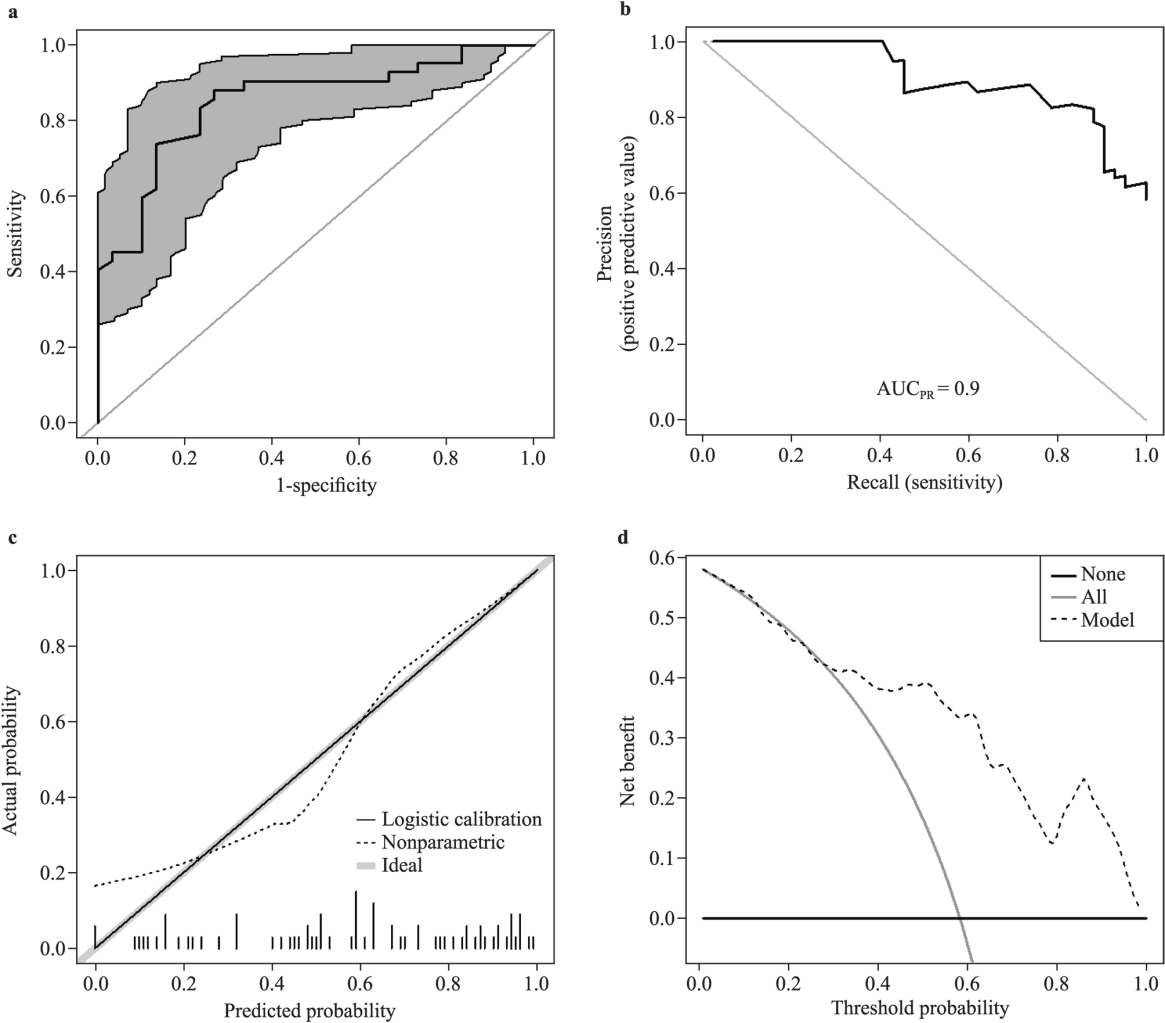
Evaluation of the model for predicting therapeutic efficacy of metoprolol in pediatric patients with vasovagal syncope and clinical utility. **a** Receiver-operating characteristic curve. Area under the curve (AUC): 0.85; sensitivity (the abscissa): 88.1%; specificity (the ordinate): 73.3%; 95% confidence interval (gray part): 0.76–0.94. The positive predictive value was 80.4%, and the negative predictive value was 80.8%; **b** precision‒recall curve. The AUC was 0.9; precision is the abscissa, and recall is the ordinate; **c** calibration plot. The abscissa is the predicted incidence of events (predicted probability), and the ordinate is the observed frequency (observed probability). The dotted line perfectly meets the diagonal if the predicted probability matches the observed probability; **d** decision curve analysis. In contrast to default strategies, decision curve analysis provides a clinical "net benefit", changing from 0.01 to 0.58

The power of the predictive model was assessed using the following methods. According to the ROC curve displayed in Fig. 4a, the area under the curve (AUC) was 0.85, and the best cut-off value was 0.5, resulting in a sensitivity of 88.1% and a specificity of 73.3%. The positive predictive value was 80.4%, and the negative predictive value was 80.8%. The AUC of the PR curve was 0.9, indicating promising predictive results (Fig. 4b). The Hosmer–Lemeshow test was used to evaluate consistency, which showed that the model fit well (*P* = 0.27). The calibration plot showed that the actual and predicted probabilities were similar (Fig. 4c). DCA revealed that the net benefit changed from 0.01 to 0.58 (Fig. 4d).

## Discussion

Our study focused on developing a therapeutic prediction model for metoprolol in pediatric patients with VVS. We found that the MPV and PDW in the treatment-effective group were greater than those in the treatment-ineffective group. Based on multivariate logistic regression, age and the MPV were used to predict therapeutic efficacy. According to the ROC analysis, PR curve, calibration plot, and Hosmer–Lemeshow test, the model demonstrated moderate predictive accuracy and a strong fit between its predictions and the observed data. According to the DCA, the predictive model could lead to improved clinical outcomes.

By comparing the baseline characteristics of the children suffering from VVS treated with metoprolol, we found that the baseline MPV and PDW in the treatment-effective group were greater than those in the treatment-ineffective group. Although there is currently no unified standard reference range for PDW in children, Hu's research pointed out that the range for PDW in a healthy control group with a median age of 12 years was 12.1% (10.8%, 13.1%) [19]. The median age of the children included in our study was also 12 years, and the PDW range was 12.4% (11.2%, 12.9%), which was similar to that in Hu’s research [19]. In our research, there was no significant difference in heart rate between the treatment-effective and treatment-ineffective groups, which was in accordance with the previous studies by Yuan et al. [20] and Kong et al. [21]. There are different phenotypes of VVS, including vasoinhibitory, cardioinhibitory, and mixed VVS. Our results showed that there was no relationship between the different phenotypes of VVS and the metoprolol efficacy. We speculated that the reason that the effective rate was similar among the patients with three phenotypes of VVS might be associated with the resting catecholamine levels. No difference in 24-hour urine NE levels was found between the vasoinhibitory and mixed subtypes of VVS in children [21], which supported our speculation.

The MPV represents the average platelet size of a blood sample. Newly generated platelets are larger than older platelets; therefore, a high MPV may imply that the bone marrow produces new platelets at an increased speed under different stimuli. The PDW reflects the variability in platelet size and is considered as a marker of platelet activation. A high PDW indicates that platelet size varies greatly, usually resulting from platelet activation and an increased number of newly generated platelets. Therefore, a high MPV and PDW are related to platelet activation and increased platelet production. VVS is known as the most common type of neurally mediated syncope [22], and increased sympathetic activity has been found in some patients with neurally mediated syncope [23], while enhanced sympathetic activity is directly related to platelet production according to several previous studies [10].

The mechanisms by which sympathetic excitation promotes platelet production and activation may be explained as follows: (1) two primary sympathetic transmitters, NE and EP, can drive platelet activation. An increase in arterial plasma EP concentrations significantly stimulates blood platelet parameters [24]. In vitro, NE mediated platelet activation in a concentration- and time-dependent manner [25]; (2) NE and EP can boost megakaryocyte attachment, movement, and proplatelet formation via adrenoceptor-mediated extracellular signal-regulated kinase stimulation, which causes a noticeable increase in platelet production [9]; (3) activation of adrenergic receptors may stimulate circulating platelets by boosting surface levels of P-selectin, enhancing conformational modifications of the glycoprotein IIb/IIIa receptor [26, 27]. These studies support the hypothesis that the increased MPV and PDW may indicate increased sympathetic activity among patients in the treatment-effective group. Furthermore, these findings partially explain the favorable response to the β-adrenergic receptor blocker metoprolol observed in our study. However, the PDW was not included in the prediction model after logistic regression, which might imply that the influence of the PDW on therapeutic outcomes was not sufficiently weighted.

Furthermore, in a recent study, proteomic analysis revealed that the expression of platelet-related proteins was upregulated in patients with postural orthostatic tachycardia syndrome (POTS), another type of orthostatic intolerance characterized by an increase in sympathetic activity [28]. Although no similar studies have been conducted in patients with VVS, the results observed in the context of POTS are consistent with our findings.

Univariate analysis also revealed that the proportion of females in the treatment-effective group was greater than that in the treatment-ineffective group. Age was included in the prediction model, which indicated that younger children with VVS may respond better to metoprolol treatment. These results suggest age- and sex-related differences in the efficacy of metoprolol. A study based on the influence of sex on heart rate variability in children revealed that female children presented significantly greater values than male children did in terms of the standard deviation of the RR intervals and absolute high frequency [29]. A large multicohort study on the development of the cardiac autonomic nervous system in children revealed that sympathetic activity decreased linearly with age, whereas parasympathetic activity increased from infancy to childhood, plateaued during middle childhood, and then decreased slightly throughout adolescence [30]. These results indicate that the development of autonomic nervous system activity varies with age and sex, which may account for the distinct responses to adrenergic receptor blockers. However, further research is needed to confirm this phenomenon and its underlying mechanisms to provide information for the treatment and management of autonomic nervous system diseases, including VVS.

All factors were included in an LASSO regression, a notable feature of which is the incorporation of the penalty term λ for variable selection in the model. It is widely used in the medical field for model construction [31, 32]. Based on the non-zero values, five variables, namely, age, duration of hospitalization, sex, MPV, and PDW, were included in the logistic regression. Based on logistic regression, the prediction model was constructed with the following equation: logit(*p*) = − 14.511 − 0.267 × age + 1.725 × MPV. This study demonstrated the feasibility and importance of nomograms for therapeutic prediction [33]. We successfully developed a nomogram based on this model. The nomogram showed satisfactory predictive ability and clinical applicability.

In numerous studies, traditional methods, including ROC curves [34], PR curves [35], calibration curves [36], and the Hosmer–Lemeshow test [37], have been used to evaluate the efficacy of prediction models. As an alternative assessment method, DCA has been widely adopted in recent research studies [38, 39]. This approach allows the calculation of the “net benefit” in clinical practice concerning prediction models [40], thereby integrating the preferences of decision-makers into the analysis. As a predictive tool, our model showed satisfactory performance at a threshold value of 0.5, with AUCs of 0.85 and 0.9 for the ROC and PR curves, respectively. According to the calibration plot and Hosmer–Lemeshow test, the model demonstrated moderate predictive accuracy and a strong fit between the predictions and observations. According to the DCA, our predictive model could lead to improved clinical outcomes, with a net improvement ranging from 0.01 to 0.58. In addition to a limited threshold probability range, treatment based on the prediction model could yield substantial benefits in comparison to treating all or none of the patients.

Although prior studies have used several factors, such as 24-hour urine NE [21], the Poincaré plot [20], baroreflex sensitivity [13], and the left-ventricular ejection fraction [41], to predict the therapeutic efficacy of metoprolol, each has restrictions in terms of cost, operability, or exponential stability. Our study provides the first nomogram based on this model to enhance its clinical applicability. A prediction model comprising age and the MPV is convenient to apply, because all the included factors are easy to collect in clinical practice. For practical reasons, we hope that this study will not only serve as a conclusion but also as a new beginning for future well-designed studies that include larger cohorts and investigate the therapeutic efficacy of metoprolol in children with VVS.

Nonetheless, our study is subject to certain limitations. Firstly, the study was limited by the use of a single hospital with an insufficient number of patients, which could introduce a potential bias that may understate the influence of factors on therapeutic efficacy. Secondly, the biomarkers were measured only at baseline; however, it may be worthwhile to continue monitoring them and examining their dynamic changes over time. Thirdly, the follow-up period was considerably short. Long-term follow-up helps gain a deeper understanding of the progress and prognosis of the disease. Furthermore, due to dysfunction of the autonomic nervous system, another limitation could be the lack of analysis of heart rate variability and NE-level measurements. We concur that further research encompassing large-scale investigations with multiple factors and robust designs is warranted to validate or refute our findings.

## Data Availability

The datasets analyzed during the current study are available from the corresponding author upon reasonable request.
